# miR-215-5p Suppresses Proliferation/Cell-Cycle Progression and Promotes Apoptosis via Targeting CTCF in Goat Mammary Epithelial Cells

**DOI:** 10.3390/ani16030484

**Published:** 2026-02-04

**Authors:** Sijiang Liu, Hongxin Sun, Manhong Wei, Jiangtao Huang, Zilong Guo, Yujie Han, Xian Qiao, Hongqiang Li, Huaiping Shi, Baolong Liu, Yuexin Shao

**Affiliations:** 1 Hebei Key Laboratory of Specialty Animal Germplasm Resources Exploration and Innovation, College of Animal Science and Technology, Hebei Normal University of Science &Technology, Qinhuangdao 066600, China; 18041373413@163.com (S.L.); 17795830654@163.com (M.W.); 17333409563@163.com (Z.G.); 15132469183@163.com (Y.H.); qiaoxian@hevttc.edu.cn (X.Q.); lihongqiang1128@163.com (H.L.); 2 College of Animal Science and Technology, Northwest A&F University, Yangling 712100, China; huaipingshi@nwafu.edu.cn; 3 Hebei Institute of Animal Science and Veterinary Medicine, Baoding 071000, China; sdlqshx@126.com; 4 State Key Laboratory of Cancer Biology, Department of Physiology and Pathophysiology, School of Basic Medical Science, Air Force Medical University, Xi’an 710032, China

**Keywords:** cell cycle, apoptosis, goat mammary epithelial cells, CTCF, miR-215-5p

## Abstract

MicroRNA (miRNA) is a type of small non-coding RNA that influences various biological processes by targeting gene expression. However, the roles of microRNA in mediating ruminant mammary cell proliferation and survival remain poorly understood. This study aimed to elucidate how miR-215-5p regulates cell cycle and apoptosis-related genes in goat mammary epithelial cells (GMECs). The effects of miR-215-5p on cell cycle and apoptosis were assessed by flow cytometry. A combination of bioinformatics analysis was conducted to predict the target genes of miR-215-5p; this was followed by experimental validation using techniques such as luciferase reporter assays. The effects of CTCF, the targeting gene of miR-215-5p, on cell cycle and apoptosis were examined by qRT-PCR, Western blot and flow cytometry in GMECs. The study demonstrated that miR-215-5p induced cell-cycle arrest at the G0/G1 phase and promoted apoptosis in GMECs. Mechanistically, miR-215-5p downregulated CTCF expression by directly targeting its 3′-untranslated region (3′UTR). This miR-215-5p-mediated depletion of CTCF inhibits CDK2 and CDK6 activity, consequently downregulating genes involved in cell-cycle progression. Furthermore, the miR-215-5p/CTCF axis was found to promote apoptosis by downregulating the protein expression of Bcl-xL and upregulating the gene expression of Bax. In summary, miR-215-5p suppresses GMEC proliferation and survival through CTCF-dependent histone modifications.

## Simple Summary

The study investigated miR-215-5p’s role in goat mammary epithelial cells (GMECs) in the context of regulating cell cycle and apoptosis. miR-215-5p induced G0/G1 arrest and apoptosis by targeting CTCF’s 3′UTR, reducing its expression. This downregulated CDK2/CDK6 and cell-cycle genes, while promoting apoptosis via Bcl-xL reduction and Bax upregulation. CTCF depletion altered histone modifications (H3K4me3, H3K27ac) at the promoters of cell-cycle/apoptosis genes. The findings reveal miR-215-5p suppresses GMEC proliferation/survival through CTCF-dependent epigenetic mechanisms; this offers insights into miRNA-mediated regulation of milk performance in dairy goats.

## 1. Introduction

Goat mammary epithelial cells (GMECs) are located in the mammary gland acini and serve as the primary sites for milk synthesis and secretion. To adapt to hormonal fluctuations and inflammatory signals during lactation, these cells undergo tightly regulated developmental processes, including proliferation, differentiation, and apoptosis [1]. Meanwhile, the proliferation, differentiation and apoptosis processes of cells enable the structural remodeling of breast tissue from its development during pregnancy to the stable state during lactation, and finally to its degeneration stage. Studies have demonstrated that both proliferation and apoptosis of mammary epithelial cells maintain a dynamic balance throughout gestation and lactation periods in lactating animals [2]. Notably, the rate of apoptosis surpasses the rate of proliferation in end-lactation [3]. Consequently, understanding the regulatory mechanisms governing GMECs’ proliferation and apoptosis is essential for enhancing milk performance during end-lactation. Research has shown that microRNAs (miRNAs) play a key role in GMECs’ proliferation and apoptosis [4,5,6].

MicroRNAs (miRNAs) play a crucial role in post-transcriptional gene regulation by binding target mRNAs and inducing mRNA degradation or inhibiting translation. Recent research has highlighted the significant impact of miRNAs on mammary gland development [7] and lactation [8]. For instance, miR-574-5p has been shown to inhibit GMEC growth and milk synthesis by targeting the ecotropic viral integration site 5 like (EVI5L) gene [9], while miR-223 suppresses apoptosis in GMECs through the RIG-I-like receptor signaling pathway [10]. Additionally, miR-214-5p binds to lactoferrin (LTF) mRNA, thereby inhibiting its expression in GMECs [11]. Zhu Lu et al. employed CRISPR/Cas9 technology to knock out miR-103 in GMECs, revealing its role in inhibiting lipid droplet, triglyceride, and cholesterol accumulation in ecotropic viral integration targeting the phospholipid scramblase 4 (PLSCR4) and pantothenate kinase 3 (PANK3) genes [12]. Studies related to cellular processes have demonstrated that mir-215-5p plays a key regulatory role in cell apoptosis [13,14]. Research by Cai et al. demonstrated that miR-215-5p is responsible for the decrease in factors associated with myocardial development and differentiation that are triggered by selenium deficiency in chickens [15]. In addition, miR-215-5p mediated the cell cycle of liver cells and the differentiation of adipocytes [16]. However, the specific function and regulatory mechanism of miR-215-5p in GMECs remain largely unexplored.

The TargetScan database has predicted that miR-215-5p targets the CTCF (CCCTC-binding factor), which is related to epigenetics [17]. CTCF, a crucial transcription factor, plays vital roles in DNA methylation patterns and regulating gene expression [18,19]. CTCF has been reported to contribute to mammary gland development. For instance, CTCF promoted the transdifferentiation of mammary preadipocytes, as evidenced by analyses of DNA methylation and protein profiles [20]. In addition, CTCF depletion promotes the expression of target genes linked to cell division, thereby accelerating the proliferation rate of breast cancer cells [21]. CTCF also plays a potential role in regulating lactation. For example, there are at least five CTCF binding sites near the whey acidic protein (WAP) site of the mammary gland super enhancer [22,23]. In addition, chromatin immunoprecipitation sequencing (ChIP-seq) analysis of breast tissue revealed the presence of four CTCF-binding domains in the casein locus [22]. Although the importance of CTCF in regulating mammary gland development and lactation has been recognized, there have been no reports as to whether miR-215-5p regulate CTCF gene expression and thereby affect the cell cycle and apoptosis of GMECs.

This study aims to elucidate the molecular mechanisms by which miR-215-5p/CTCF regulates the apoptosis and proliferation of GMECs. The findings of this study provide a novel approach to the regulatory role of miRNAs/transcription factor in mammary gland development and lactation in dairy goats.

## 2. Materials and Methods

### 2.1. Animal Care

The study protocol related to the animals (protocol code 18–602) was approved by the Institutional Animal Care and Use Committee at Northwest A&F University.

### 2.2. Cell Culture

Samples from the mammary glands of healthy three-year-old Xinong Saanen dairy goats, which were in various stages of lactation—namely dry, early, peak, and late—were collected at intervals of 15, 60, 120, and 270 days postpartum (*n* = 5). For the extraction and pooling of GMECs, three Xinong Saanen dairy goats in the peak lactation phase were selected. The previously established protocols for the isolation, purification, characterization, and culture of GMECs were utilized [24]. In brief, mammary tissue was surgically obtained and subsequently rinsed with D-Hank’s solution. The tissue was then cut into approximately 1 mm^3^ cubes, placed in 60 mm dishes, and incubated in a 5% CO_2_ environment at 37 °C. The culture medium was refreshed every two days until epithelial cells detached from the tissue blocks. Following this, cells were digested from the tissue using a 0.25% trypsin−ethylenediaminetetraacetic acid (EDTA) solution. To purify the GMECs, the fibroblasts were removed by a differential adhesion technique. Finally, the suspended GMECs, along with the culture medium, were moved to new culture dishes. The cells were maintained in an incubator set at 37 °C with an atmosphere of 5% CO_2_ throughout the experiment. The culture medium used consisted of DMEM/F12 medium (Gibco, Carlsbad, CA, USA), supplemented with 10% fetal bovine serum (FBS, Zlife, Grand Island, NY, USA), penicillin/streptomycin (10,000 units/L, Harbin Pharmaceutical Group, Harbin, China), and epidermal growth factor (1 mg/L, PHG0311, Invitrogen Corp., Carlsbad, CA, USA).

### 2.3. Vector Construction

Primers for sequence cloning of the goat CTCF gene (GenBank no. XM_018062160.1) are described in Table S1. The cloning of the gene was generated as described elsewhere [25]. For the synthesis of cDNA, 500 ng of total RNA was treated with the gDNA Eraser enzyme to eliminate DNA contamination, using the PrimeScript RT Reagent Kit (4368814, Invitrogen, CA, USA) with gDNA Eraser. Hind III and Not I restriction sites were suitable for the cloning process. The cDNA from the GMECs provided the coding sequence template and the 3′UTR region for the CTCF cloning. The goat CTCF coding sequence and pcDNA3.1 vector were used to construct a vector, pcDNA3.1-CTCF. The psi-WT-CTCF vector was created, which encompasses the CTCF 3′UTR in the psiCHECK2 vector. For the construction of the site-directed mutant vector psi-MUT-CTCF of 3′UTR of CTCF, overlapping PCR was used. The sequences of all constructed vectors were analyzed through sequencing.

### 2.4. Cell Treatment

The TargetScan database revealed that miR-215-5p targets the 3′UTR of CTCF. Treatments commenced when the cells reached 80% confluence. To induce the overexpression of miR-215-5p, cells were transfected with either the miR-215-5p mimic or the negative control mimic NC (50 nM, RiboBio, Guangzhou, China), using Lipofectamine RNAiMAX (13778150, Invitrogen Corp., Carlsbad, CA, USA). Following this, the GMECs were treated with the miR-215-5p inhibitor or its negative control inhibitor NC (100 nM, RiboBio, Guangzhou, China) to facilitate the knockdown of miR-215-5p. CTCF gene overexpression was achieved by transfecting cells with the pcDNA3.1-CTCF vector via Lipofectamine Lip3000 (L3000075, Invitrogen Corp., Carlsbad, CA, USA). The silencing of the CTCF gene in GMECs was performed using specific small interference RNA (si-CTCF, GenePharma, Shanghai, China). Table S2 contains the sequences for si-CTCF and the negative control si-NC. There were three replicates per condition for each group.

### 2.5. Cell Immunofluorescence Technic

Cells were cultured in 12-well plates (*n* = 3 per group). For the detection of the signal and localization of CTCF protein, a miR-215-5p mimic was treated in the cells for 48 h. After treatment, the cells were washed and fixed in 4% (*w*/*v*) paraformaldehyde. After fixation, the cells were stained with anti-CTCF antibody (ab128873, abcam) at a dilution of 1:100. It is worth noting that cells were washed three times with PBS and incubated with CTCF protein antibody overnight at 4 °C. The subsequent day, the cells were washed for three cycles in PBS at room temperature for a period of 40 min; this was followed by incubation in secondary antibody for more than 2 h. Following this, the cells were rinsed with PBS and DAPI staining. Following staining, the sections were rinsed with PBS three times for five minutes and then promptly treated for an additional five minutes with a reagent that quenches spontaneous fluorescence. After that, they were washed with distilled water for 10 min. Ultimately, images were obtained with the help of fluorescence microscopy.

### 2.6. Flow Cytometry

Flow cytometry analysis measured the distribution and cell apoptosis of the cell cycle. Cells were cultured in 6-well plates (*n* = 3 per group). The experimental treatments were carried out when cell confluence reached 80%. After 48 h treatment, GMECs were collected for the cell-cycle and apoptosis assay. A Cell Cycle Staining Kit (SeaBiotech, Shanghai, China) and flow cytometer were utilized to detect the cell cycle. The cells were harvested and were washed three times with PBS. Then, 75% ethanol in PBS fixed the cell overnight at −20 °C. The apoptosis of the GMECs was tested with the flow cytometry method and an Annexin V-FITC PI staining apoptosis assay kit (SeaBiotech, Shanghai, China).

### 2.7. Quantitative Real-Time PCR Assay (qRT-PCR)

RNAiso Plus (9109, Takara, Otsu, Japan) was utilized to extract the total RNA from the cells, following the previously outlined methods [26,27]. cDNA was synthesized for gene quantitative analysis according to RNA reverse transcription kit methods (G3337-100, Servicebio, Wuhan, China). The SYBR Green kit from Beijing Qingke Biotechnology Co., Ltd., Beijing, China was utilized for qRT-PCR. The gene selected as a control was ubiquitously expressed transcript (UXT). Gene expression analysis primers are presented in Table S3. For the synthesis of cDNA related to mature mir-215-5p, the miRcute cDNA kit (KR211, Tiangen, Beijing, China) was utilized for quantitative analysis. The quantification of miR-215-5p levels was carried out using the miRcute q-PCR kit (FP411, Tiangen) through the qRT-PCR method. The quantitative primer sequence of miR-215-5p was 5′-CGCGGTGCGCATGACCTATGAATTGAC-3′. The qRT-PCR experiments were conducted using the Bio-Rad CFX96 analyzer (Hercules, CA, USA). Quantitative data were assessed using the 2^−ΔΔCt^ method.

### 2.8. Western Blot Assay

Cells were cultured in 6-well plates (*n* = 3 per group). Cells were lysed using RIPA lysis reagent (R0010, Solarbio, Beijing, China) for Western blotting analysis. Following the methodology of a previous study, a BCA assay kit was employed to determine protein concentrations [28]. The protein samples were subjected to electrophoresis using 10% SDS-polyacrylamide gels, after which they were transferred onto a nitrocellulose membrane (HATF00010, Millipore, Burlington, MA, USA). To block nonspecific binding sites, 5% skim milk (232100, BD Biosciences, Franklin Lakes, NJ, USA) was applied for a minimum of 2 h. Then, samples were incubated with the primary antibody: rabbit CDK2 (R22532, diluted at 1:1000, Zen-Bioscience, Chengdu, China), rabbit CDK6 (R23891, diluted at 1:1000, Zen-Bioscience, Chengdu, China), CTCF (ab128873, diluted at 1:1000, abcam), PCNA (R25293, diluted at 1:1000, Zen-Bioscience, Chengdu, China) or Bcl-xL (R23603, diluted at 1:1000, Zen-Bioscience, Chengdu, China). The secondary antibody was horseradish peroxidase (HRP)-conjugated goat anti-mouse-IgG or goat anti-rabbit-IgG (CW0102 or CW0103, CW Biotech, Jiangsu, China).

### 2.9. Assay for Transposase-Accessible Chromatin with High Throughput Sequencing (ATAC-Seq)

Cells were plated in 60 mm culture dishes for ATAC-seq. Nuclei were purified from frozen GMEC samples. A total of six samples were used to construct libraries for ATAC-seq. The nuclei of cells in three replicates in the NC and CTCF groups were lysed using the High-Sensitivity Open Chromatin Profile Kit 2.0 (N248, Suzhou Nearshore Protein Technology Co., Ltd., Suzhou, China). ATAC-seq was performed as previously described [29]. Briefly, after going through steps such as cell collection and lysis, transposase fragmentation, magnetic bead recovery of fragmented DNA, and library amplification and magnetic bead sorting, the DNA fragments are ultimately transformed into libraries suitable for sequencing. The evaluation of the constructed libraries’ quality was conducted utilizing the Agilent 2100 Bioanalyzer platform (Agilent Technologies, Santa Clara, CA, USA). Following this, high-throughput sequencing was executed on the Illumina NovaSeq 6000 system (Illumina, San Diego, CA, USA) at Gene Denovo Biotechnology Co., Ltd. (Guangzhou, China). FastQC were used for the quality control of the raw sequence reads from all samples. Then, Fastp (v0.19.11) software was used to remove adapter sequences and poor-quality reads. BWA (Burrows-Wheeler Alignment) (v0.7.17) was used to map clean reads to the goat reference genome. Opening or closing peaks were identified as |log2(fold change)| ≥ 0.5 and *p* < 0.05. During the genome-wide annotation of peaks, the functional regions were divided into promoter, downstream, exon, intron and distal intergenic regions. DeepTools was used to visualize the ATAC-seq signals [30,31].

### 2.10. CUT TAG (Cleavage Under Targets and Tagment)

Cells were plated in 60 mm culture dishes for cellular CUT TAG. The process of CUT TAG sequencing involved the estimation of traction of CTCF and histone H3 lysine 4 trimethylation (H3K4me3) and H3 lysine 27 acetylation (H3K27ac) substances on the indicated genes in order to study their effects on CDK2 and CDK6 gene transcription. The manufacturers’ instructions were followed to perform CUT TAG (N259-YH01, Suzhou Nearshore Protein Technology Co., Ltd., Suzhou, China). In short, cells were placed in 100 mm dishes and subjected to the applicable treatment at the correct time. Cells were treated with quenching solution (10× glycine solution) after being fixed with 1% formaldehyde for cross-linking of DNA and proteins at 37 °C for 10 min. A sonication process was carried out at 4 °C to lyse the cells and shear the DNA to an average length of 150 to 400 base pairs. The DNA was preserved for the subsequent assay. The chromatin solution underwent a preclearance process utilizing protein A/G beads and was subjected to immunoprecipitation with the H3K4me3 antibody (1:100, 502357, Chengdu Zhengneng Biotechnology Co., Ltd., Chengdu, China) by incubation overnight at 4 °C. The washes were performed by washing sequentially with wash buffers and eluting with TE Buffer.

### 2.11. Statistical Analysis

All experiments were repeated at least for 3 biological replicates. Data are shown as mean ± SEM and were analyzed using the SPSS 20.0 software (IBM, Chicago, IL, USA). A T-test was utilized for comparison of data between pairs of groups, and one-way analysis of variance with the Tukey test was carried out for multiple comparisons. The difference was significant when *p* < 0.05 (* *p* < 0.05 and ** *p* < 0.01).

## 3. Results

### 3.1. The Impact of miR-215-5p on the Cell Cycle and Apoptosis in GMECs Has Been Observed

**Figure 1:**
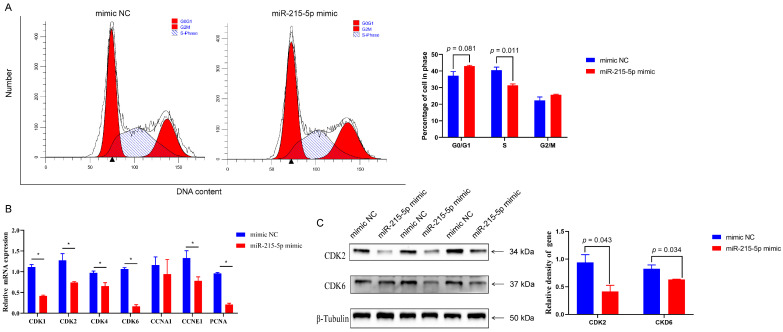
Overexpression of MiR-215-5p suppressed GMEC proliferation. (**A**) Cell cycle of GMECs treated with miR-215-5p mimic. (**B**) Expression of gene related to cell cycle in GMECs treated with miR-215-5p mimic (100 nM) for 24 h (* *p* < 0.05). (**C**) Expression of the CDK2 and CDK6 genes in GMECs was detected by Western blot after treatment with miR-215-5p mimic (100 nM) for 48 h (* *p* < 0.05).

**Figure 2:**
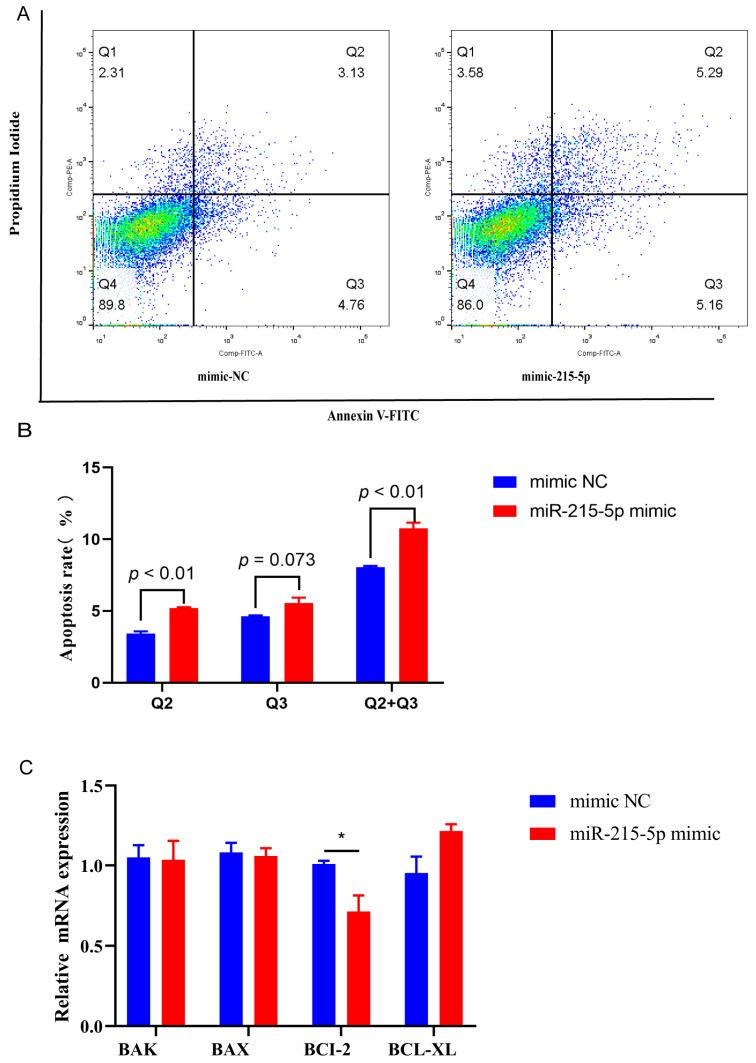
Susceptibility analysis of the GMECs activated by overexpression of miR-215-5p. (**A**) Detection of apoptosis using annexin V-FITC and PI staining assay through flow cytometry. (**B**) Statistical analysis of the apoptotic rate. (**C**) Expression of BAK, Bax, BCL-2 and Bcl-xL gene in GMECs treated with miR-215-5p mimic (100 nM) for 24 h (* *p* < 0.05).

Flow cytometry analysis revealed that the transfection of mimics of miR-215-5p led to an increase in the quantity of GMECs in the G0/G1 phase over a 48 h period. (Figure 1A). miR-215-5p led to a reduction in the quantity of GMECs in the S phase over a 48 h period. (Figure 1A). Moreover, the expression levels of the CDK1, CDK2, CDK4, CDK6, CCNE1, and PCNA genes were found to be lowered after miR-215-5p transfection (Figure 1B). According to Western blot analysis, CDK2 and CDK6 protein levels were decreased by miR-215-5p (Figure 1C). The staining using FITC/PI revealed the significant increase in early and late apoptotic cells in the experimental group that was treated with miR-215-5p mimic (Figure 2A,B). Moreover, treatment with miR-215-5p caused a reduction in the level of B-cell lymphoma-2 (BCL-2), which is a gene linked to apoptosis (Figure 2C).

### 3.2. Targeted Genes and Functions of miR-215-5p

**Figure 3:**
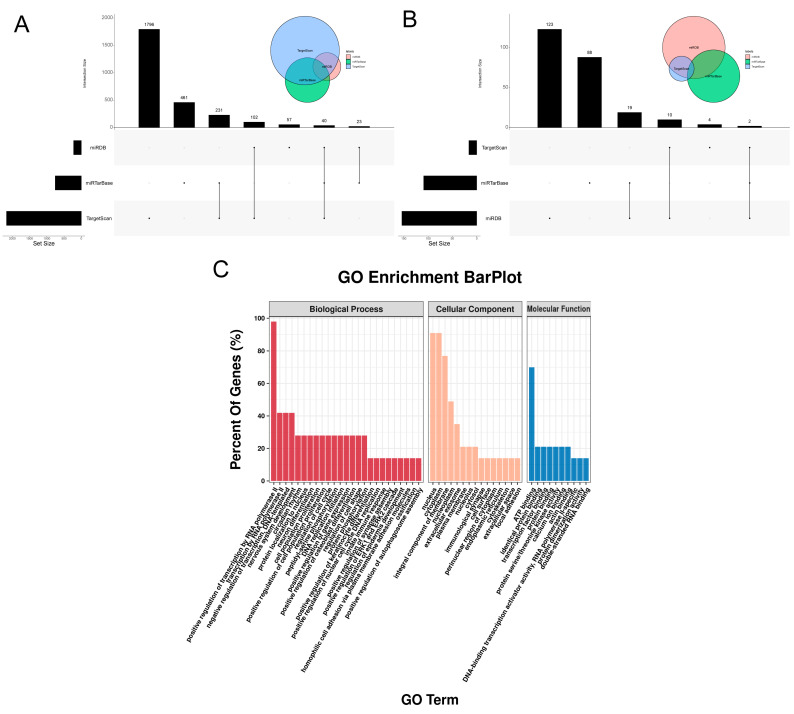
Prediction of miR-215-5p target genes. (**A**) MicroRNA regulation of CTCF expression, as rendered by prediction software. (**B**) miR-215-5p target gene prediction Venn diagram. (**C**) miR-215-5p target gene analysis by GO analyze.

The mature sequence seed region UGACCU of miR-215-5p encoded by goats, through comparative analysis of miR-215-5p sequences of various species, was found to be the same as those of five other species, including humans, rats, and macaques (Table 1). This finding indicates that miR-215-5p is highly conserved throughout the process of species evolution. The prediction of the target genes and functions of miR-215-5p was conducted using TargetScan, miRTarBase, and miRDB. It was determined that miR-215-5p regulates the expression of 40 genes, including CTCF (Table S4 and Figure 3A).

Conversely, to screen the potential binding miRNAs on the CTCF’s UTR region, the above prediction tools were utilized again. The results showed that miR-215-5p and miR-221-3p can bind to the human CTCF gene (Figure 3B), which is an important regulator of cell proliferation and differentiation [32]. However, only miR-215-5p can bind to the 3′UTR region of the goat CTCF, and thus, it was selected for further GO enrichment analysis. The primary enriched terms for biological process (BP), cellular component (CC), and molecular function (MF) among the 40 targeted genes were, respectively, the positive regulation of transcription by RNA polymerase II, the nucleus, and ATP binding. (Figure 3C).

### 3.3. miR-215-5p Targets 3′UTR of CTCF

To investigate the regulatory roles of miR-215-5p on CTCF in the mammary gland during different lactation stages in goat, we measured their relative RNA levels in the four key stages. The miR-215-5p level was highest during the dry-lactation stage, followed by a marked decrease during the early-lactation stage (*p* = 0.083 compared to dry-lactation, Figure S1). Notably, the miR-215-5p level showed a significant difference between the dry-lactation and peak-lactation stages (*p* = 0.032, Figure S1). CTCF expression exhibited an opposite pattern compared to miR-215-5p. CTCF expression during the early-lactation stage is lower than that during the dry-lactation stage (*p* < 0.01).

**Figure 4:**
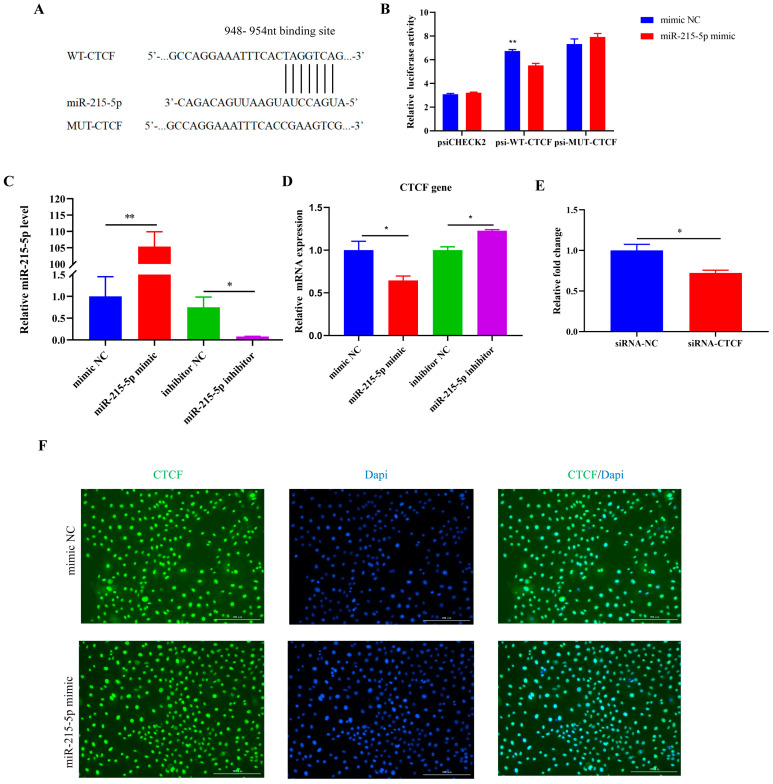
miR-215-5p targets 3′UTR of *CTCF* gene. (**A**) Predicted binding site of miR-215-5p in *CTCF* 3′UTR and construction of the wild-type sequence vector (psi-WT-CTCF) and the mutation vector (psi-MUT-CTCF). (**B**) 3′UTR activity of CTCF was measured by luciferase assay. (**C**) miR-215-5p expression in GMECs was measured. (**D**) Expression of the *CTCF* gene in GMECs treated with miR-215-5p mimic (50 nM) or miR-215-5p inhibitor (100 nM). (**E**) Expression of the *CTCF* gene in GMECs treated with miR-215-5p mimic (50 nM) by immunofluorescence. (**F**) Immunofluorescence staining of CTCF. DAPI, blue; CTCF, green. * *p* < 0.05 and ** *p* < 0.01, compared with control.

MiR-215-5p might possibly target the goat CTCF gene, based on the prediction of the TargetScan database (Figure 4A). To investigate whether miR-215-5p directly targets the 3′UTR of CTCF, we examined the dual-luciferase activity of wild-type and mutant CTCF upon transfection of miR-215-5p (Figure 4B). When the potential binding site of miR-215-5p on the CTCF gene was mutated, there was no significant difference observed in the dual-luciferase activity after transfecting miR-215-5p (Figure 4B), indicating the sequence is essential for the binding of the miRNA.

Next, we examined the transfection efficiency of miR-215-5p. Compared with negative control miRNA (mimic NC), the expression of miR-215-5p in the miR-215-5p-treated group was up-regulated 105 times. Nevertheless, the expression of miR-215-5p was down-regulated in the miR-215-5p inhibitor-treated cells (Figure 4C). The inhibitory effects of miR-215-5p on CTCF were then measured. The gene expression in the miR-215-5p group was significantly reduced, by 40%, but it was upregulated upon miR-215-5p inhibitor treatment (Figure 4D). In addition, the Western blot results showed that overexpression of miR-215-5p decreased the CTCF protein level (Figure S2). The result of immunofluorescence staining revealed that overexpression of miR-215-5p decreased the expression of CTCF, as quantified in Figure 4E,F. These results demonstrated that miR-215-5p directly binds to the specific sequence on the 3′ UTR of CTCF, thus regulating its expression in GMECs.

### 3.4. Effects of CTCF Expression on Cell Cycle and Cell Apoptosis

**Figure 5:**
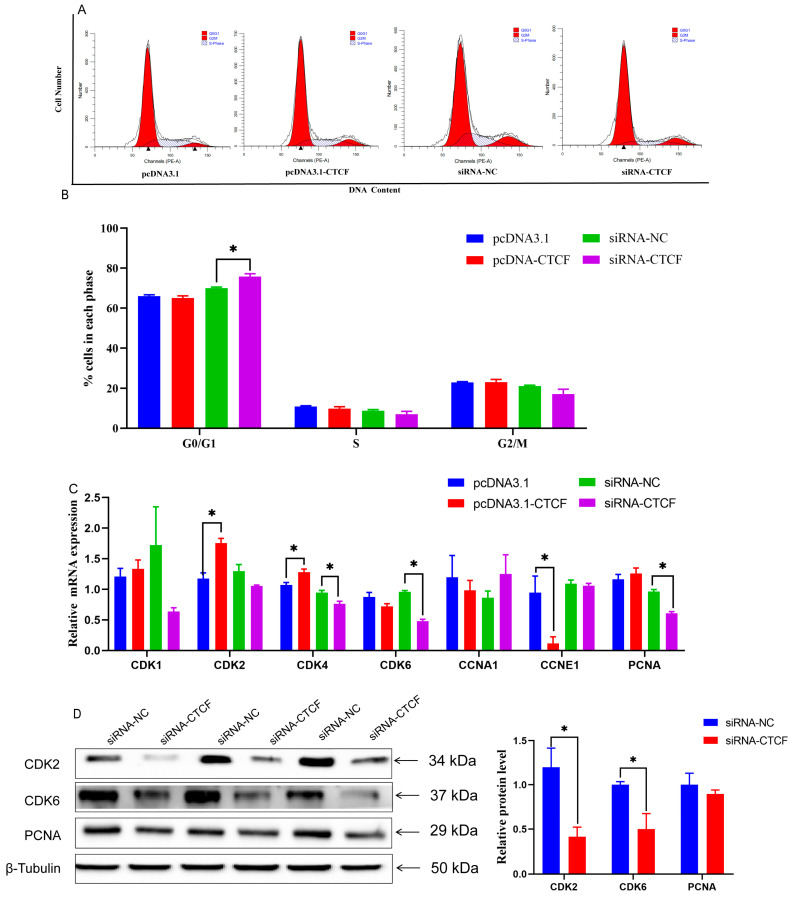
Effects of CTCF overexpression and knockdown in GMECs. (**A**) Cell cycle of GMECs. (**B**) Cell numbers in the different phases. (**C**) Expression of gene related to cell cycle and cell apoptosis. (**D**) Expression of CDK2, CDK6 and PCNA in GMECs was detected by Western blot. Data are presented as mean ± SEM. * *p* < 0.05 compared with control.

To investigate whether CTCF affects cell proliferation, we measured cell cycle and cell apoptosis with flow cytometry (Figure 5A). The results showed that overexpressing CTCF had no impact on cell-cycle progression. In contrast, siRNA aimed at CTCF resulted in a notable halt of the cell cycle during the G0/G1 phase (*p* < 0.05, Figure 5B). Furthermore, CTCF overexpression promoted the transcription of genes such as CDK2, CDK4, and CCNE1. Conversely, in cells transfected with CTCF-specific siRNA (siCTCF), the expression levels of CDK4, CDK6, and PCNA were markedly suppressed (*p* < 0.05, Figure 5C). Western blot analysis confirmed that reduced CTCF expression led to a significant decrease in the protein levels of CDK2 and CDK6 (*p* < 0.05, Figure 5D).

**Figure 6:**
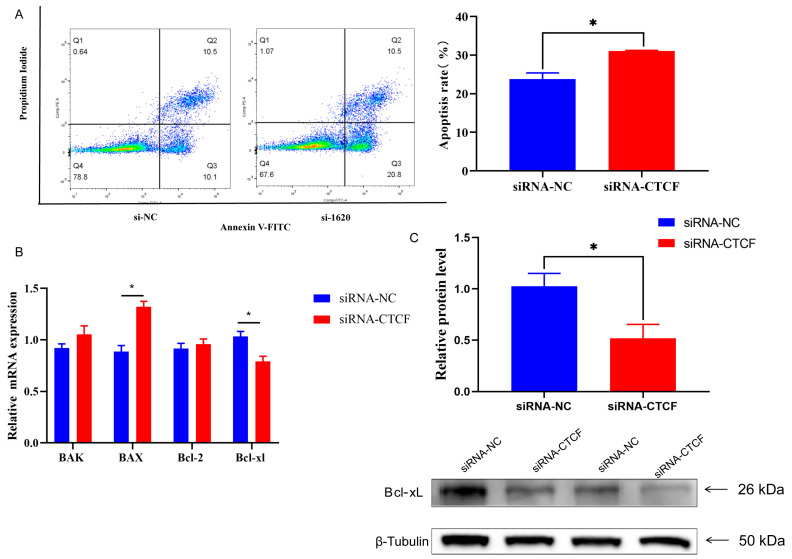
Effects of CTCF knockdown in cell apoptosis of GMECs. (**A**) Rate of cell apoptosis. (**B**) Expression of gene related to cell apoptosis. (**C**) Western blotting of Bcl-xL protein expression in cells. Data are presented as mean ± SEM. * *p* < 0.05 compared with control.

We then examined how CTCF influences cell apoptosis. The results from FITC/PI staining indicated that there was an increase in the number of apoptotic cells within the CTCF-knockdown group when compared to the control group (*p* < 0.05, Figure 6A). Following this, we assessed the expression levels of Bcl-xL, a protein from the BCL-2 family that functions as an anti-apoptotic factor. The results showed that knocking down CTCF markedly downregulated the mRNA levels of Bcl-xL (*p* < 0.05, Figure 6B). Additionally, knocking down CTCF expression activated the transcription of the Bax gene (*p* < 0.05, Figure 6B). Moreover, Western blot results showed that knocking down CTCF expression decreased Bcl-xL protein levels (*p* < 0.05, Figure 6C). Collectively, these results underscore the pivotal role of CTCF in modulating cell proliferation and cell apoptosis through alteration of the apoptotic gene expression.

### 3.5. Knockdown of CTCF Reduces the H3K4me3 and H3K27ac Levels in the Promoter Regions of the Target Genes

**Figure 7:**
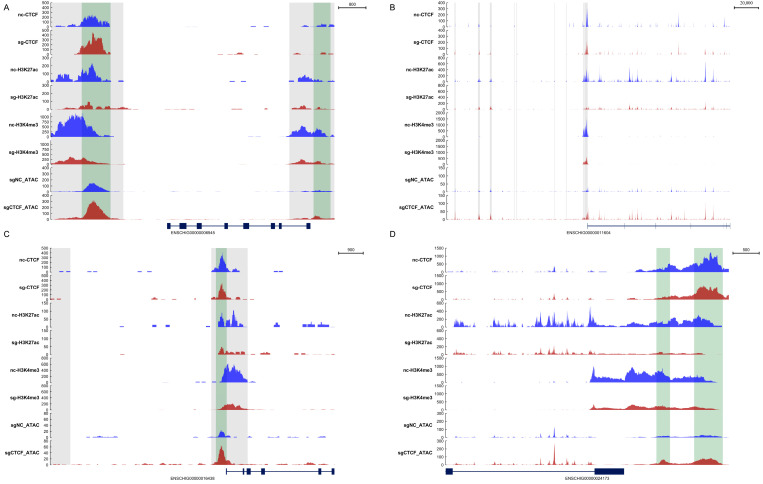
Identification of cell cycle- and cell apoptosis-regulated genes in CTCF-deficient GMECs. (**A**) Genomic snapshot of the CDK2 locus. (**B**) Genomic snapshot of the CDK6 locus. (**C**) Genomic snapshot of the Bax locus. (**D**) Genomic snapshot of the Bcl-xL locus. Densities of CUT TAG-seq reads for CTCF, H3K27ac, and H3K4me3 are shown. Densities of ATAC-seq reads are also shown.

To gain insight into how CTCF regulates those cell cycle- and apoptosis-related genes, including CDK2, CDK4, Bax, and Bcl-xL, we explored genome-wide analysis of CTCF recruitment. We aimed to investigate the interaction of CTCF with the promoters of target genes, using ATAC-seq and CUT TAG-seq techniques. The ATAC-seq results indicated that knockdown of CTCF increased the chromatin opening degree of the CDK2, CDK4, Bax, and Bcl-xL genes (Figure 7). Next, the CUT TAG-seq data for H3K4me3 and H3K27ac showed characteristic patterns around the CTCF binding regions. H3K4me3 was the histone modification most frequently found at active promoters. CTCF knockdown significantly reduced the enrichment of both H3K4me3 and H3K27ac at the CDK2 locus and CDK4 locus (Figure 7A,B). Notably, the loss of H3K27ac at the CDK2 promoter correlated with its decreased expression (Figure 7A). Similarly, the results revealed a marked depletion of these active histone marks near the Bax and Bcl-xL promoters in CTCF-depleted cells (Figure 7C,D). These findings suggest that CTCF plays a regulatory role in the activity of promoters associated with genes linked to the cell cycle and apoptosis.

**Figure 8:**
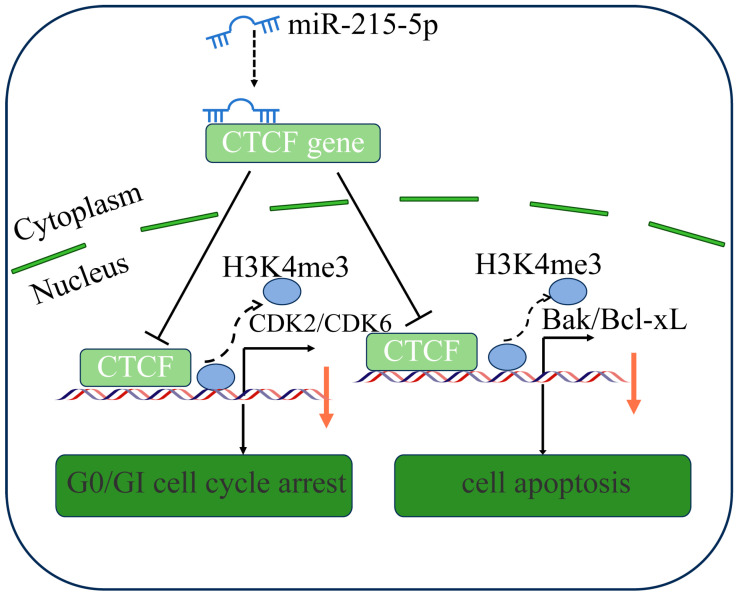
The proposed model regarding the role and regulation of miR-215-5p in goat mammary epithelial cells. miR-215-5p inhibits CDK2 and CDK6 expression by targeting 3′UTR of the CTCF gene. Then, CTCF interacts with histone H3K4me3 and is involved in the regulation of CDK2 and CDK6. Moreover, the interaction between CTCF and these cyclin-dependent kinases (CDKs) is shown to be associated with the G0/G1 cell-cycle arrest.

Overall, our study revealed that miR-215-5p affects cell cycle and apoptosis by regulating the expression of the CTCF gene in GMECs. miR-215-5p arrests cells at the G0/G1 phase via the CTCF-CDK2/CDK6 axis and promotes cell apoptosis via the CTCF-Bax/Bcl-xL axis (Figure 8).

## 4. Discussion

The amount and composition of milk in dairy goat operations is affected by the developmental stage of the mammary gland and the quantity of mammary epithelial cells present. In this work we evaluated the effects of miR-215-5p on GMEC proliferation and apoptosis and elucidated the mechanisms involved. The binding of mir-215-5p to the 3′ UTR of the CTCF gene inhibits its expression, leading to a halt in the G0/G1 phase of the cell cycle and an increase in apoptosis within GMECs.

Studies have shown that goat mammary gland development and milk production are regulated by multiple functional genes, transcription factors, and small non-coding RNA [33,34,35]. miR-215 belongs to the miR-192 family, known for its significant conservation and critical function in cellular proliferation [36,37]. The regulation function of miR-215-5p in the context of milk lactation is unknown, despite its potent features. During the early stage of lactation, GMECs exhibit heightened proliferative activity, which promotes the development of mammary alveoli and the dilation of milk secretion ducts. In contrast, this proliferative activity markedly diminishes during the end stage of lactation. This research examined the expression levels of miR-215-5p in dairy goats throughout various stages of lactation, using qRT-PCR. We discovered that the mRNA levels of miR-215-5p were notably diminished during early lactation compared to late lactation. This observation suggests that miR-215-5p may be involved in regulating the cell cycle of GMECs, consequently influencing milk production throughout different lactation stages. Our results demonstrated that miR-215-5p inhibited cell proliferation and increased the cell apoptosis rate, as revealed by the cell flow cytometry experiment (Figure 1A). Additionally, we observed downregulation of key cell-cycle regulatory genes upon miR-215-5p treatment, including CDK2 and CDK6, which are crucial for the G1 to S phase transition. Our findings show that the functions of miR-215-5p in regulating GMEC proliferation and apoptosis are comparable to its roles in other types of cells. However, the detailed molecular mechanisms remain unclear.

It has been reported that miRNA-215-5p could negatively regulate the expression of RUNX1, and its overexpression induced apoptosis and consequently inhibited cellular proliferation in multiple-myeloma cells [38]. The prediction made by the TargetScan database (http://www.targetscan.org/) indicates that miR-215-5p interacts with the 3′UTR region of the CTCF gene in goats. Additionally, to verify the direct interaction between miR-215-5p and the CTCF gene, a dual-luciferase assay was conducted (Figure 4). Importantly, the expression levels of the CTCF gene were reduced by miR-215-5p. Consequently, these findings imply that CTCF plays a role in mediating the impacts of miR-215-5p on the inhibition of cell proliferation and the facilitation of apoptosis in GMECs. CTCF is a zinc-finger protein with roles in chromatin architecture, gene expression, and cell-cycle control [39]. In studies on the development of skeletal muscle and neurological development, CTCF has been recognized as an essential candidate gene involved in the proliferation and differentiation of various cell types [40,41]. This study utilized overexpression and interference techniques to examine the influence of CTCF on genes associated with the multiple-myeloma cell cycle and apoptosis in dairy goats. Flow cytometry analyses demonstrated that interference with CTCF in GMECs increased the G0/G1 phase population in GMECs, potentially through the regulation of CDK2, CDK4, CDK6, and PCNA (Figure 5). In a previous study, CDK2, a critical component that modulates the cell cycle, has been revealed as a targeted gene of CTCF [42]. In complex with cyclin E (G1 phase) and cyclin A (S phase), CDK2 contributes greatly to the mammary epithelial cell proliferation during breast tumorigenesis [43]. CDK2 phosphorylates the retinoblastoma protein (RB) at multiple sites (e.g., Ser567, Ser608), thus releasing E2F transcription factors to activate S-phase genes (MCM7, CDC6) [44,45]. In mammary gland development, this pathway is tightly regulated by CDK4 and CDK6 during puberty and pregnancy [46]. And in this study, CDK4, CDK6, and PCNA were found to be targeted by CTCF as well. Together, our findings suggest that CTCF is a master transcriptional factor that governs the cell cycle, and one which is negatively regulated by miRNA-215-5p in GMECs.

Previous research has indicated that miR-215-5p may promote apoptosis in breast cancer cells by interacting with the 3′UTR of RAD54B, thereby influencing cell viability and genomic stability [47]. Another study on breast cancer cells revealed that CTCF triggers transcriptional silencing of p53, thus facilitating cell survival and tumorigenesis [48]. In our study, CTCF has been identified as a direct target of miR-215-5p. However, how this axis regulates GMECs’ survival was unclear. Our data reveal that miR-215-5p downregulated the apoptosis-related gene BCL-2 and promoted apoptosis in GMECs (Figure 2A). Moreover, the reduction of CTCF in GMECs initiates early apoptosis, which is marked by an increase in the expression of the pro-apoptotic gene Bax and a decrease in the expression of the anti-apoptotic gene Bcl-xL (Figure 6B,C). In addition, consistent with results in other cellular types, we found that CTCF predominantly binds to the proximal promoter region of Bax in GMECs, suggesting a conserved mechanism of transcriptional control [49]. Therefore, the miR-215-5p/CTCF axis exerts a pro-apoptosis effect in GMECs. As for the different targeted apoptosis-related genes, a plausible explanation is that miR-215-5p also binds to other critical genes (in addition to CTCF) to induce apoptosis, such as RAD54B and RUNX1, as predicted (Table S4). In a previous study, Bax, a pivotal pro-apoptotic protein that regulates cell apoptosis, was identified as a targeted gene of CTCF [50]. As a central anti-apoptotic regulator within the Bcl-2 family, Bcl-xL exerts its effects by heterodimerizing with pro-apoptotic members via its BH3-binding groove [51]. Taken together, these observations underscore the critical role of the miR-215-5p/CTCF axis in regulating cellular survival and apoptosis in GMECs. In future studies, we will conduct rescue experiments to demonstrate that the effect of miR-215-5p is mediated by CTCF.

Furthermore, this study indicated that CTCF modulates the chromatin accessibility in the promoter regions of cell cycle- and apoptosis-related genes, as revealed by ATAC-seq. Alterations in the level of chromatin accessibility within the promoter region can influence various processes, including cell proliferation and apoptosis. CTCF–cohesin loops physically disrupt the interaction between H3K27ac-marked enhancers and CDK2 and CDK6 promoters, thereby abolishing transcriptional activation. In senescent fibroblasts, CTCF-mediated looping silences CDK2 by sequestering its promoter from an active SE [52]. In this study, CTCF depletion reduced the proximity of CDK2 and CDK6 promoters to distal regulatory elements, thereby silencing their expression in GMECs. In CTCF-knockdown GMECs, in which the expression of Bcl-xL and Bax is very low, we found near-baseline levels of H3K27ac and H3K4me3 at the Bcl-xL and Bax loci. We speculated that CTCF expression influences the levels of methylation and acetylation within the promoter areas of genes associated with apoptosis, including Bcl-xL and Bax. H3K4me3 and H3K27ac serve as counteracting epigenetic marks at the CDK2/CDK6 loci, with their balance dictating transcriptional output [53]. Broad H3K4me3 peaks at the promoter regions of CDK2 and CDK6 correlate with their high basal transcription levels [54]. H3K27ac-marked super-enhancers upstream of CDK2 and CDK6 in cancer cells drive their overexpression [55]. These findings collectively suggest that CTCF acts as a central hub in the regulatory network governing cell-cycle progression and apoptosis in GMECs.

## 5. Conclusions

This study provides evidence for the miR-215-5p/CTCF axis in the regulation of cell cycle and apoptosis in GMECs. Specifically, CTCF enhances the chromatin accessibility and induces histone modifications such as H3K4me3 and H3K27ac at the promoter regions of genes associated with cell cycle and apoptosis, thereby promoting the proliferation and survival of GMECs. However, miR-215-5p could suppress CTCF by targeting its 3′ UTR region, thus reversing the effects. This finding offers molecular insights into the regulatory mechanisms of the miR-215-5p/CTCF axis that governs the balance between proliferation and apoptosis in GMECs.

## Figures and Tables

**Table 1:** The conservation analysis ofmiR-215-5p.

Serial Number	Species	miRNA Name	Conservative Sequence
MIMAT0036060	*Capra hircus*	>chi-miR-215-5p	AUGACCUAUGAAUUGACAGAC
MIMAT0003118	*Rattus norvegicus*	>rno-miR-215	AUGACCUAUGAUUUGACAGAC
MIMAT0000272	*Homo sapiens*	>hsa-miR-215-5p	AUGACCUAUGAAUUGACAGAC
MIMAT0000904	*Mus musculus*	>mmu-miR-215-5p	AUGACCUAUGAUUUGACAGAC
MIMAT0002728	*Macaca mulatta*	>mml-miR-215-5p	AUGACCUAUGAAUUGACAGAC
MIMAT0001134	*Gorilla gorilla*	>gga-miR-215-5p	AUGACCUAUGAAUUGACAGAC

## Data Availability

Dataset available on request from the authors.

## Supplementary Materials

The following supporting information can be downloaded at: https://www.mdpi.com/article/10.3390/ani16030484/s1, Figure S1: CTCF gene and miR-215-5p abundance in goat mammary gland of different lactation stages; Figure S2: Expression of CTCF in cells treated with miR-215-5p mimic; Table S1: Primers used for amplification of CTCF; Table S2: Sequences of siRNA targeting goat CTCF gene; Table S3: Primers used for quantitative real-time polymerase chain reaction of genes; Table S4: miR-215-5p target genes shared by prediction software.
