# Supplementary material for: miR-215-5p Suppresses Proliferation/Cell-Cycle Progression and Promotes Apoptosis via Targeting CTCF in Goat Mammary Epithelial Cells
Source: Animals (Basel). 2026 Feb 4;16(3):484. doi: 10.3390/ani16030484 (PMC12897051; doi:10.3390/ani16030484)

Supplemental Table S1. Primers used for amplification of CTCF

primer name	primer sequence (5′-3′)
CTCF(CDS clone)	F:CCCAAGCTTATGGAAGGTGAGGCGGTTGA
	R:CCGCTCGAGTCACCGGTCCATCATGCTGAG
CTCF (3'UTR clone)	F: CCGCTCGAGGCAGTGTAAGAAAACCCAGCA
	R: ATAAGAATGCGGCCGCGTCTCATGTTGGCGTCCTAA
CTCF (3'UTR mutagenesis)	F: CAGGAAATTTCACCGAAGTCGTAATT
	R: AATTACGACTTCGGTGAAATTTCCTG

Supplemental Table S2. Sequences of siRNA targeting goat CTCF gene

siRNA name	sense sequence (5'-3')	antisense sequence (5'-3')
si-NC	UUCUCCGAACGUGUCACGUTT	ACGUGACACGUUCGGAGAATT
si-CTCF	GCACUUCAAACGCUAUCAUTT	AUGAUAGCGUUUGAAGUGCTT

Supplemental Table S3. Primers used for quantitative real-time polymerase chain reaction of genes

gene	primer sequence (5′-3′)
CFCF	F: GATTGCTGACCAGAGGCTTGA<br>R: TCCATTTCCCCTTCCTCTCCGT
CDK1	F: CCAATAATGAAGTGTGGCCAGAAG<br>R: AGAAATTCGTTTGGCAGGATCATAG
CDK2	F: GAGTCGGATCGCAACTTGGA<br>R: CTCTCGGCTGCTGCATTGT
CDK4	F:TTTGCTGAGATGGTGACCCG<br>R: TAACTCCTGGCCAAACCACC
CDK6	F: GATGGGTTTCCACCAGGGAG<br>R: CTTTGTAACCCGATGCTGCG
CCNA1	F: AAGTAGACACCAGCGCACTC<br>R: GGCCTGTGTCTTATTTCGGC
CCNE1	F: CTCCCTGATTCCCACACCTG<br>R: CATAAGATGCTTGTCCCTCA
PCNA	F: CGCTTAAGGATCTCATCAATGAG<br>R: GTTACGGTCGCAGCGGTAAG
BAK	F: TCTCCCCGAGAGGTCTTTTT<br>R: TGATGGTCCTGATCAACTCG
BAX	F: ATGTGTGTGGAGAGCGTCAA<br>R: GTCCAATGTCCAGCCCATGA
BCL-2	F: ATGTGTGTGGAGAGCGTCAA<br>R: CTAGGGCCATACAGCTCCAC
BCL-XL	F: TGACCACCTAGAGCCTTGGA<br>R: AAGAGTGAGCCCAGCAGAAC
UXT	F: TGTGGCCCTTGGATATGGTT<br>R: GGTTGTCGCTGAGCTCTGTG

Supplemental Table S4. miR-215-5p target genes shared by prediction software.

Prediction software shared miR-215-5p target genes			
WNK1	ENC1	RAD54B	MCM10
ALCAM	CTCF	STX7	SLC19A2
KHDRBS3	SIX4	EMC7	PIP4K2B
NAB1	B3GALNT1	DICER1	NSF
RPAP2	PABPC4	NCOA3	NOD2
ATF1	DYRK1A	DDX50	CCDC47
OSBPL10	FGF2	CDC7	KIF5B
FNDC3B	MSN	LIMS1	FAXC
PHTF2	CLSTN1	TOR1AIP1	IGDCC4
EML6	CEP85L	ID4	RUNX1

Supplemental Figure S1

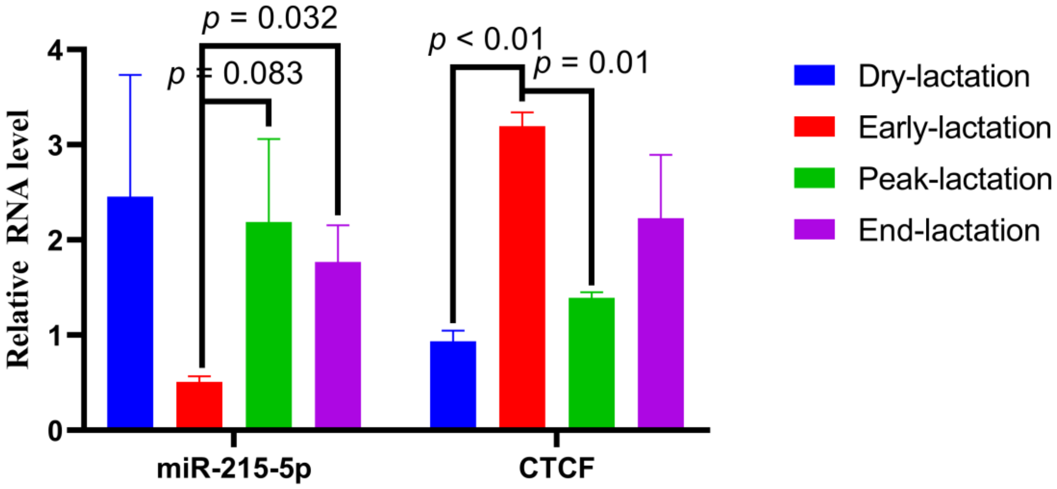

Supplemental Figure S2

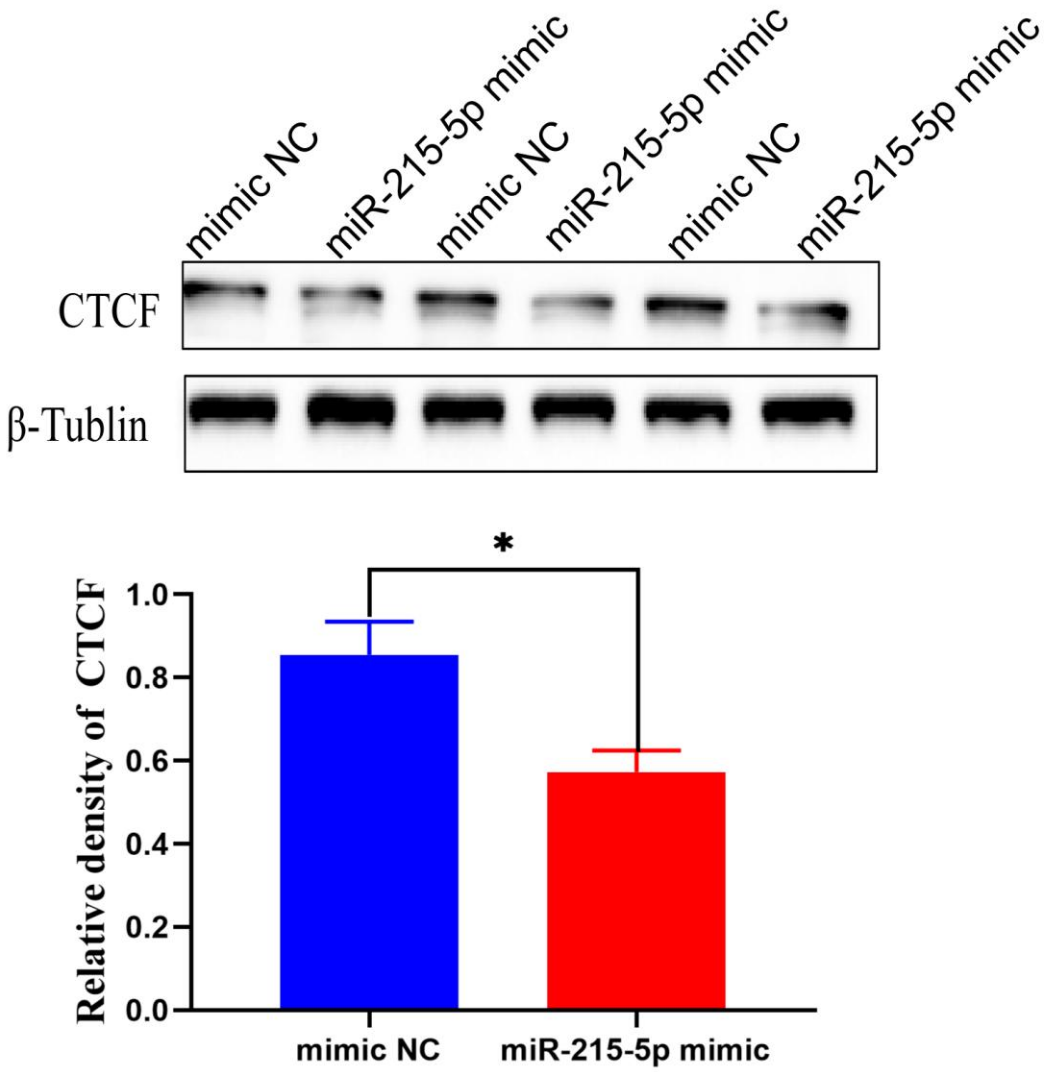

Supplement: Supplementary file 1 [file animals-16-00484-s001.zip › animals-4049836-supplementary.pdf]
